# Prasugrel effectively reduces the platelet reactivity units in patients with genetically metabolic dysfunction of cytochrome P450 2C19 who are treated with long-term dual antiplatelet therapy after undergoing drug-eluting stent implantation

**DOI:** 10.1007/s00380-019-01499-7

**Published:** 2019-09-23

**Authors:** Junichiro Shimamatsu, Ken-ichiro Sasaki, Yoshio Katsuki, Tomohiro Kawasaki, Yoshinobu Murasato, Hidehiko Ajisaka, Hiroyoshi Yokoi, Hideki Tashiro, Atsushi Harada, Yuji Hirakawa, Yuta Ishizaki, Takashi Ishimatsu, Kotaro Kagiyama, Yoshihiro Fukumoto, Tatsuyuki Kakuma, Takafumi Ueno

**Affiliations:** 1 Division of Cardiovascular Medicine, Department of Internal Medicine, Kurume University School of Medicine, 67 Asahimachi, Kurume, 830-0011 Fukuoka Japan; 2 Division of Cardiovascular Internal Medicine, Tanushimaru Chuo Hospital, Kurume, Japan; 3 Division of Cardiology, Sugi Hospital, Omuta, Japan; 4 Department of Cardiology, Cardiovascular Center, Shin Koga Hospital, Kurume, Japan; 5 Department of Cardiology, National Hospital Organization Kyushu Medical Center, Fukuoka, Japan; 6 Division of Cardiovascular Internal Medicine, Asakura Medical Association Hospital, Asakura, Japan; 7 Division of Cardiovascular Internal Medicine, Fukuoka Sanno Hospital, Fukuoka, Japan; 8 Division of Cardiovascular Internal Medicine, St. Mary’s Hospital, Kurume, Japan; 9 Daiichi Sankyo Co., Ltd., Tokyo, Japan; 10 Biostatistics Center, Kurume University, Kurume, Japan

**Keywords:** Percutaneous coronary intervention, Clopidogrel, Genetic polymorphism, Drug change

## Abstract

Dual antiplatelet therapy (DAPT) with aspirin and P2Y_12_ inhibitor is administered following percutaneous coronary intervention (PCI) with coronary stent implantation. Several studies have reported the effects of switching between P2Y_12_ inhibitors on platelet reactivity (P2Y_12_ reaction units: PRU), from acute to late phase after PCI. However, the effect of switching at very late phase is unknown. This study examined the effect on PRU in Japanese coronary heart disease patients with long-term DAPT (aspirin + clopidogrel) when switching from clopidogrel to prasugrel. Ninety-six patients were enrolled in this study. The median DAPT duration at enrollment was 1824.0 days. Twenty-three patients with PRU ≥ 208 at enrollment were randomly assigned into either continuing to receive clopidogrel (Continued Group; *n* = 11) or switching to prasugrel (Switched Group; *n* = 12). The primary endpoint was the rate of patients who achieved PRU < 208 at the end of 12 weeks of treatment, which was significantly higher in Switched Group relative to Continued Group (90.0% vs. 36.4%; *P* = 0.024). The secondary endpoint was the PRU at week 12 in groups subdivided according to cytochrome P450 (CYP) 2C19 genotypes. At week 12, extensive metabolizers (EM Group) had 202.3 ± 60.0 and 174.5 ± 22.3 in Continued Group and Switched Group (*P* = 0.591), respectively; intermediate and poor metabolizers (non-EM Group) had 229.4 ± 36.9 and 148.4 ± 48.4 in Continued Group and Switched Group (*P* = 0.002), respectively. The PRU for non-EM Group was significantly reduced in Switched Group. Thus, for patients with long-term DAPT (aspirin + clopidogrel) after PCI with coronary stent implantation, switching from clopidogrel to prasugrel resulted in a stable reduction in PRU, regardless of CYP2C19 polymorphism.

## Introduction

The guidelines recommend dual antiplatelet therapy (DAPT) with aspirin and P2Y_12_ inhibitor to prevent major adverse cardiovascular events, such as stent thrombosis and reinfarction, in patients after percutaneous coronary intervention (PCI) with coronary stent implantation [1–3]. Clopidogrel has been commonly used as a P2Y_12_ inhibitor for DAPT.

However, it is also reported that the genetic polymorphisms of its metabolizing enzyme, cytochrome P450 (CYP) 2C19, are involved in the antiplatelet effect of clopidogrel [4, 5]. The CYP2C19 genotypes are classified based on rate of drug-metabolizing activity into three groups: extensive metabolizers (EM), intermediate metabolizers (IM), and poor metabolizers (PM), causing inter-individual variability in response to clopidogrel [6, 7]. In addition, prevalence of IM and PM in the Japanese population is higher than in Western populations [6, 8].

The P2Y_12_ reaction units (PRU), an index of antiplatelet effects of P2Y_12_ inhibitors, are also reported to be a relevant factor associated with cardiovascular events [9, 10]. In the latest guidelines, the routine testing of platelet function when using antiplatelet agents is not recommended (Class III) with Level of Evidence A [2]. However, the ADAPT-DES study reported that the incidence of stent thrombosis or myocardial infarction was significantly higher in patients with PRU > 208 after successful PCI, compared to patients with PRU ≤ 208 [11, 12]; and, considering such, the platelet function testing may not as insignificant as claimed.

In the studies that examined association of PRU with CYP2C19 polymorphism in patients treated with clopidogrel, PRU in IM and PM patients was reported significantly higher than in EM patients [7, 13, 14]. Additionally, frequency of cardiovascular events was reported higher in patients treated with clopidogrel who carry CYP2C19 loss-of-function alleles, compared to patients without the alleles [15, 16]. These reports suggest that, even in patients receiving DAPT (aspirin + clopidogrel) after coronary stent implantation, possible future cardiovascular events could be predicted by PRU measurement or CYP2C19 genotype test to identify patients with inadequate antiplatelet response.

Prasugrel is known to be less affected by CYP2C19 polymorphism [17]. The PRASFIT-ACS [18, 19] and PRASFIT-Elective [20] conducted in Japan have reported that prasugrel steadily lowered PRU and reduced the incidence of cardiovascular events in patients irrespective of the CYP2C19 polymorphism. Recent studies have reported that switching between P2Y_12_ inhibitors affects PRU [21–23]. However, never reported was the change in PRU in patients with different CYP2C19 polymorphism when switching P2Y_12_ inhibitors from clopidogrel to prasugrel in patients receiving long-term DAPT (aspirin + clopidogrel).

Several reports have suggested that cardiovascular events occurred in spite of long-term DAPT (aspirin + clopidogrel) [12, 24]. The patients receiving long-term DAPT (aspirin + clopidogrel) with CYP2C19 polymorphism may experience the result of poor inhibition of platelet aggregation, even in the chronic phase, thereby increasing the risk of cardiovascular events. Thus, we aimed to examine the effects of switching from clopidogrel to prasugrel on PRU in patients having received long-term DAPT (aspirin + clopidogrel) after PCI with coronary stent implantation, along with CYP2C19 genotype test results.

## Methods

### Study design

This multicenter, randomized, open-label, parallel-group comparison study was conducted at eight sites in the Kyushu region, Japan, from April 2017 to August 2018. An ethical review committee at each site approved the conduct of this study. The study was conducted in accordance with the Declaration of Helsinki, ethical guidelines for clinical research, ethical guidelines for human genome/gene analysis research, and the Act on Protection of Personal Information, and followed the ICH-GCP guidelines. Monitoring and auditing were also conducted by a third-party organization to ensure the reliability of the data. The present study was registered at the University Hospital Medical Information Network Clinical Trials Registry (UMIN000027089) in Japan.

### Patient selection

Patients who met all the following inclusion criteria were enrolled: (1) underwent coronary stent implantation and were consistently with DAPT (aspirin + clopidogrel) for 52 weeks or more; (2) 20 years of age or older; (3) have provided written consent to participate; (4) provided consent on collection and analysis of samples for genetic analysis; and (5) can understand the nature of the study and follow its procedures, in the opinion of the principal investigator.

Patients were excluded if any of following criteria were met (1) bleeding tendencies or diathesis; (2) severe hepatic impairment; (3) severe renal impairment; (4) poor blood pressure control during antihypertensive therapy; (5) history of cerebral infarction or transient ischemic attack; (6) history of hypersensitivity to thienopyridine drugs; (7) pregnant, suspected to be pregnant, wish to be pregnant, or lactating; (8) those the investigator has determined are unable to provide sufficient understanding and cooperation due to mental incapacity (including moderate and severe dementia); (9) scheduled, or must be hospitalized during the observation period, at the discretion of the investigator, or were hospitalized during the period from providing consent to determining eligibility (except when hospitalized for tests); (10) must receive treatment with prohibited concomitant drugs during observation period; and (11) others judged ineligible by investigator.

### Treatment

After confirming eligibility of coronary heart disease patients who underwent coronary stent implantation and were consistently with DAPT (aspirin + clopidogrel) for 52 weeks or more, written informed consent was obtained from all enrolled in the study.

At enrollment, characteristics of these patients were aggregated as baseline data, PRU was measured using VerifyNow^®^ assay (Instrumentation Laboratory, Bedford, MA, USA), and CYP2C19 genotype test was performed. The CYP2C19 genotype test results were disclosed to all doctors and patients after completion of this study. Patients with baseline PRU ≥ 208 were randomly assigned (1:1) via an interactive web response system (IWRS), and by a minimization method using PRU as an allocation adjustment factor, into either continuing to receive clopidogrel (hereinafter “Continued Group”) or switching to prasugrel (hereinafter “Switched Group”). The PRU cutoff value was set at 208, in line with ADAPT-DES study [11, 12]. Continued Group remained on the same dosing regimen. Duration of treatment was 12 weeks, during which aspirin (81–100 mg/day) was co-administered in both groups. PRU was measured at the end of 12 weeks of treatment. The following drugs were contraindicated for co-administration during treatment period: (1) aspirin (excluding its use as basal therapy) and other antiplatelet agents, (2) oral anticoagulants, and (3) drugs prohibited from concomitant use specified in the package inserts.

### Endpoints

The primary endpoint was rate (%) of patients who achieved PRU < 208 at the end of 12 weeks. The secondary endpoints were as follows: proportion of DAPT score; examine change in PRU in Continued and Switched Groups from baseline to week 12, as well as according to CYP2C19 polymorphism (EM and non-EM (IM and PM) Groups); incidence of bleeding and cardiovascular events; and PRU at baseline according to CYP2C19 polymorphism.

### Statistical analysis

A two-sided test with significance level of 5% was used to calculate a 95% confidence interval.

### Sample size

Based on the previous study [25], the rate of patients who achieved PRU < 208 in the groups continued on prasugrel and switched to clopidogrel was 94.3% and 69.0%, respectively. Assuming a similar rate in this study and using detection power of 80% and significance level of 5%, the required number of patients was 41 per group. In consideration of dropouts, the target sample size of this study was determined as 90 patients (45 patients per group).

### Primary analysis

The primary analysis was evaluated using a Fisher’s exact test to compare rates of patients, between Continued and Switched Groups in the full analysis set (FAS), who achieved PRU < 208 at the end of 12 weeks.

### Secondary analysis

Baseline characteristics (including age, gender, medical history, and complications) for all enrolled patients were provided according to PRU (PRU ≥ 208 or PRU < 208), a *t* test or Fisher’s exact test was performed to compare the groups. Patients assigned to Continued and Switched Groups in the FAS were compared in the same manner. In the FAS, a *t* test was performed for comparison of change (± SD) in PRU from baseline to week 12, between these two treatment groups, as well as among CYP2C19 polymorphism (EM and non-EM Groups). Additionally, incidence of cardiovascular events was calculated according to these groups in the FAS. Incidence of bleeding events was also calculated in the safety analysis set (SAF), which is further aggregated according to the Bleeding Academic Research Consortium (BARC) criteria. PRU in all patients were compared according to CYP2C19 polymorphism (EM, IM, and PM).

## Results

### Study population

**Fig. 1:**
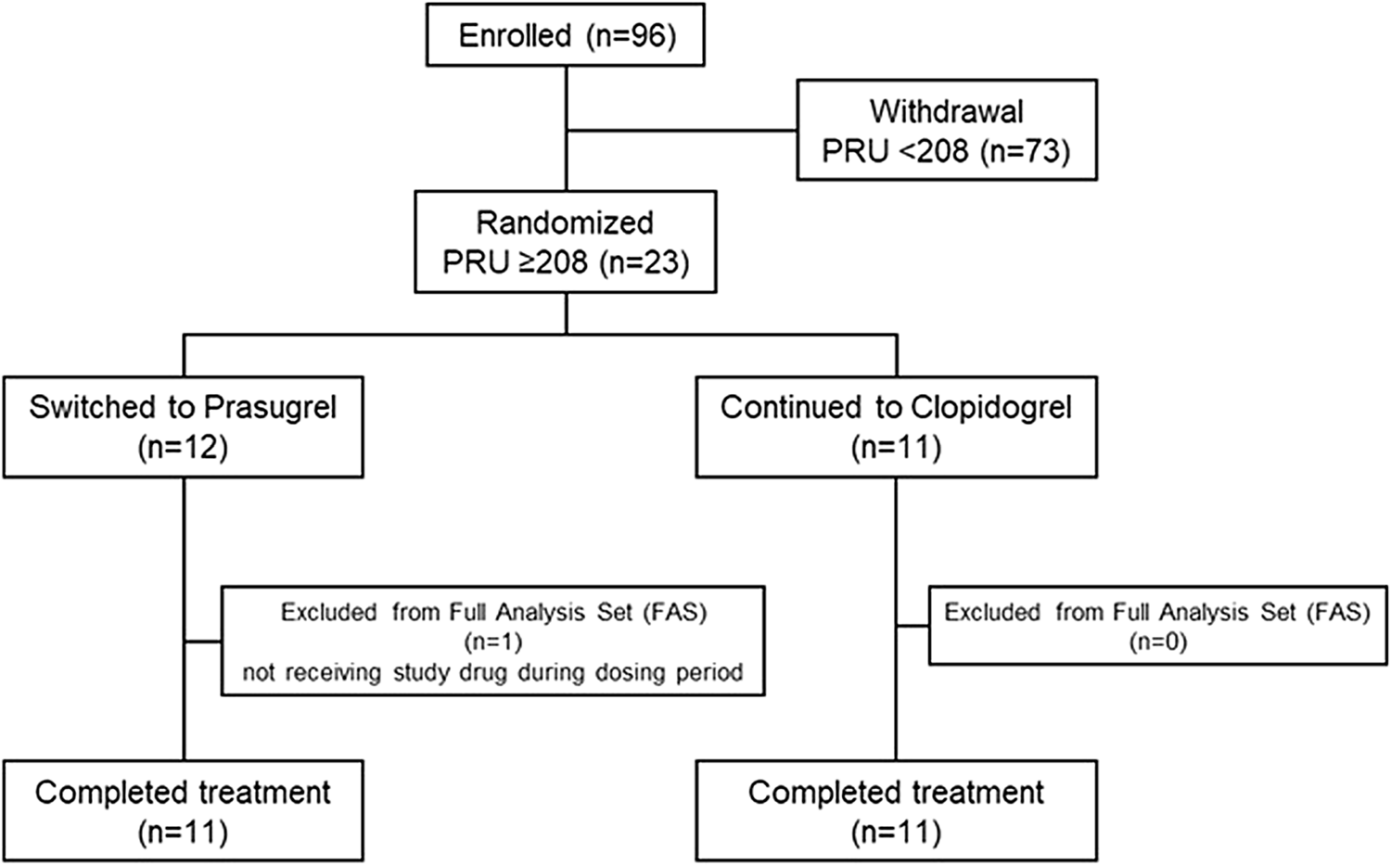
Study flowchart

**Fig. 2:**
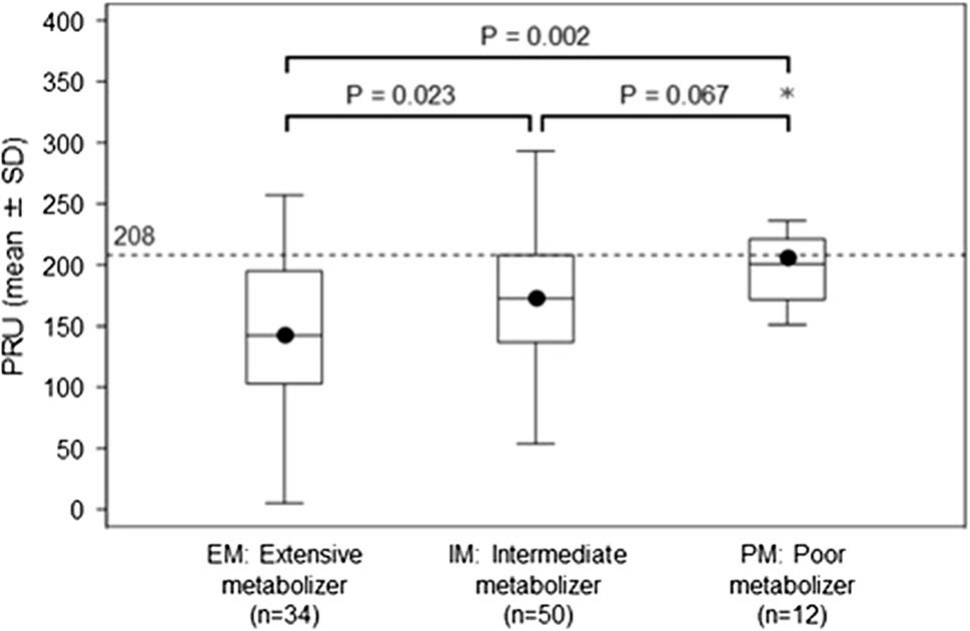
PRU at baseline by CYP2C19 polymorphism. Black dots (•) represent the mean of PRU on each metabolizers. In a box plot, central line/upper edge line represents median/the third quartile/the first quartile. Upper end of the whisker: the maximum value not exceeding the third quartile + 1.5 × interquartile range (IQR). Asterisk (*) indicates an outlier (value that is larger than the upper end of the whisker or smaller than the end of the whisker)

The flowchart of patients through the study is shown in Fig. 1. A total of 96 patients were enrolled, of which 73 (approximately 76%) had PRU < 208, and 23 (approximately 24%) had PRU ≥ 208. The patients who had PRU ≥ 208 were randomly assigned into either Continued Group (*n* = 11) or Switched Group (*n* = 12), and 11 in each group completed the 12 weeks of treatment. All in Continued Group received clopidogrel 75 mg daily. In Switched Group, 10 patients received prasugrel 3.75 mg daily, and 1 patient received 2.5 mg daily. Baseline patient characteristics are shown in Tables 1 and 2. As shown in Table 1, overall median DAPT duration was 1824.0 days (range 375–3603) after PCI with coronary stent implantation, and 55 patients (57.3%) had a DAPT score of ≥ 2. The median duration of treatment with clopidogrel was 1866.0 days (range 161–3473). The CYP2C19 genotype test identified 34 patients (35.4%) as EM, 50 (52.1%) as IM, and 12 (12.5%) as PM. The baseline PRU according to CYP2C19 polymorphism was 143.5 ± 59.7 in EM, 173.3 ± 56.3 in IM and 206.4 ± 49.8 in PM, indicating that the PRU were significantly higher in IM and PM, compared to EM. Though the PRU in PM was higher than that in IM, there was no statistically significant difference (Fig. 2). When patients with PRU ≥ 208 were compared to those with PRU < 208, the non-EM group demonstrated higher proportion, 78.3% and 60.3%, respectively, and included more patients of advanced age, females, lower height and lower body weight. In addition, fewer patients with a history of unstable angina were in the group with PRU ≥ 208, compared to those with PRU < 208. With respect to Table 2, no difference was observed other than a high percentage in Continued Group with previous myocardial infarction.

**Table 1:** Baseline characteristics

	Overall *n* = 96	PRU < 208 *n* = 73	PRU ≥ 208 *n* = 23	*p* value
Gender, *n* (%)				
Male	75 (78.1)	62 (84.9)	13 (56.5)	0.008
Female	21 (21.9)	11 (15.1)	10 (43.5)	
Age, years				
Mean ± SD	69.9 ± 9.6	68.6 ± 9.9	74.0 ± 7.3	0.020
Median [range]	71.0 [42–89]	69.0 [42–89]	74.0 [60–89]	
Height, cm				
Mean ± SD	161.73 ± 7.22	162.58 ± 7.17	159.00 ± 6.84	0.038
Median [range]	162.95 [144.6–173.0]	164.50 [144.6–173.0]	158.50 [147.8–172.5]	
BMI, kg/m^2^				
Mean ± SD	25.06 ± 3.27	25.40 ± 3.49	23.96 ± 2.16	0.064
Median [range]	24.90 [15.1–36.4]	25.42 [15.1–36.4]	24.06 [20.3–27.5]	
Weight, kg				
Mean ± SD	65.76 ± 10.74	67.36 ± 11.11	60.70 ± 7.69	0.009
Median [range]	65.10 [33.5–91.0]	68.70 [33.5–91.0]	61.60 [44.7–77.2]	
Smoking history, *n* (%)				
Never	47 (49.0)	32 (43.8)	15 (65.2)	0.095
Yes	49 (51.0)	41 (56.2)	8 (34.8)	
Medical history, *n* (%)				
Myocardial infarction	54 (56.3)	42 (57.5)	12 (52.2)	0.810
Unstable angina	25 (26.0)	23(31.5)	2 (8.7)	0.032
Ischemic stroke	0 (0.0)	0 (0.0)	0 (0.0)	–
Hemorrhage intracranial	0 (0.0)	0 (0.0)	0 (0.0)	–
Arteriosclerosis	23 (24.0)	17 (23.3)	6 (26.1)	0.784
Stable angina pectoris	30 (31.3)	20 (27.4)	10 (43.5)	0.197
Complication, *n* (%)				
Hypertension	91 (94.8)	69 (94.5)	22 (95.7)	1.000
Dyslipidemia	90 (93.8)	67 (91.8)	23 (100.0)	0.330
Diabetes mellitus	58 (60.4)	44 (60.3)	14 (60.9)	1.000
Hepatic function disorder	3 (3.1)	2 (2.7)	1(4.3)	0.565
Renal function disorder	11 (11.5)	6 (8.2)	5 (21.7)	0.126
Atrial fibrillation	1 (1.0)	0 (0.0)	1 (4.3)	0.240
Clopidogrel dose, mg				
Mean ± SD	74.7 ± 2.6	74.7 ± 2.9	75.0 ± 0.0	0.577
Median [range]	75.0 [50–75]	75.0 [50–75]	75.0 [75–75]	
Duration of DAPT, days				
Mean ± SD	1784.7 ± 834.0	1793.9 ± 847.3	1757.6 ± 811.3	0.858
Median [range]	1824.0 [375–3603]	1696.0 [375–3603]	1866.0 [404–3473]	
Duration of Clopidogrel administration, days				
Mean ± SD	1785.4 ± 838.8	1797.0 ± 846.6	1751.2 ± 833.2	0.823
Median [range]	1866.0 [161–3473]	1866.0 [346–3436]	1866.0 [161–3473]	
PRU at baseline				
Mean ± SD	166.9 ± 59.8	144.1 ± 47.0	239.3 ± 31.7	< 0.001
Median [range]	165.0 [5–341]	155.0 [5–207]	228.0 [208–341]	
CYP2C19 polymorphism, *n* (%)				
EM	34 (35.4)	29 (39.7)	5 (21.7)	0.228
IM	50 (52.1)	36 (49.3)	14 (60.9)	
PM	12 (12.5)	8 (11.0)	4 (17.4)	
IM + PM	62 (64.6)	44 (60.3)	18 (78.3)	0.139^a^
DAPT score, *n* (%)				
< 2	32 (33.3)	22 (30.1)	10 (43.5)	0.301
≥ 2	55 (57.3)	44 (60.3)	11 (47.8)	

An independent *t* test was used for measurement data, and a Fisher’s exact test was performed for count data

*EM* extensive metabolizer, *IM* intermediate metabolizer, *PM* poor metabolizer

^a^Compared with EM

**Table 2:** Baseline characteristics by treatment group (PRU ≥ 208)

	Switched to prasugrel *n* = 11	Continued clopidogrel *n* = 11	*p* value
Gender, *n* (%)			
Male	6 (54.5)	6 (54.5)	1.000
Female	5 (45.5)	5 (45.5)	
Age, years			
Mean ± SD	76.0 ± 8.0	71.7 ± 6.6	0.187
Median [range]	74.0 [64–89]	72.0 [60–80]	
Height, cm			
Mean ± SD	159.84 ± 7.50	158.38 ± 6.70	0.637
Median [range]	158.50[151.3–172.5]	160.60[147.8–166.3]	
BMI, kg/m^2^			
Mean ± SD	23.74 ± 2.49	24.33 ± 1.88	0.533
Median [range]	23.30[20.3–27.5]	24.66[20.3–26.6]	
Weight, kg			
Mean ± SD	60.69 ± 8.04	61.25 ± 7.83	0.869
Median [range]	60.40 [50.0–77.2]	62.00 [44.7–71.6]	
Smoking history, *n* (%)			
Never	7 (63.6)	7 (63.6)	1.000
Yes	4 (36.4)	4 (36.4)	
Medical history, *n* (%)			
Myocardial infarction	3 (27.3)	9 (81.8)	0.030
Unstable angina	2 (18.2)	0 (0.0)	0.476
Ischemic stroke	0 (0.0)	0 (0.0)	–
Hemorrhage intracranial	0 (0.0)	0 (0.0)	–
Arteriosclerosis	4 (36.4)	1 (9.1)	0.311
Stable angina pectoris	7 (63.6)	3 (27.3)	0.198
Complication, *n* (%)			
Hypertension	10 (90.9)	11 (100.0)	1.000
Dyslipidemia	11 (100.0)	11 (100.0)	–
Diabetes mellitus	7 (63.6)	7 (63.6)	1.000
Hepatic function disorder	1 (9.1)	0 (0.0)	1.000
Renal function disorder	2 (18.2)	3 (27.3)	1.000
Atrial fibrillation	0 (0.0)	0 (0.0)	–
Clopidogrel dose, mg			
Mean ± SD	75.0 ± 0.0	75.0 ± 0.0	–
Median [range]	75.0 [75–75]	75.0 [75–75]	
Duration of DAPT, days			
Mean ± SD	1942.6 ± 882.7	1558.5 ± 764.9	0.288
Median [range]	1937.0 [591–3473]	1505.0 [404–3051]	
Duration of Clopidogrel administration, days			
Mean ± SD	1941.5 ± 884.5	1546.4 ± 810.3	0.288
Median [range]	1937.0 [591–3473]	1505.0 [161–3051]	
PRU at baseline			
Mean ± SD	238.2 ± 37.9	238.2 ± 26.7	1.000
Median [range]	227.0 [208–341]	228.0 [208–293]	
CYP2C19 polymorphism, *n* (%)			
EM	2 (18.2)	3 (27.3)	1.000
IM	7 (63.6)	6 (54.5)	
PM	2 (18.2)	2 (18.2)	
IM + PM	9 (81.8)	8 (72.7)	1.000^a^
DAPT score			
< 2	7 (63.6)	2 (18.2)	0.070
≥ 2	3 (27.3)	8 (72.7)	
Dose after randomization, mg			
Mean ± SD	3.636 ± 0.377	75.0 ± 0.0	–
Median [range]	3.750 [2.50–3.75]	75.0 [75–75]	

An independent *t* test was used for measurement data, and a Fisher’s exact test was performed for count data

*EM* extensive metabolizer, *IM* intermediate metabolizer, *PM* poor metabolizer

^a^Compared with EM

### Primary endpoint

**Fig. 3:**
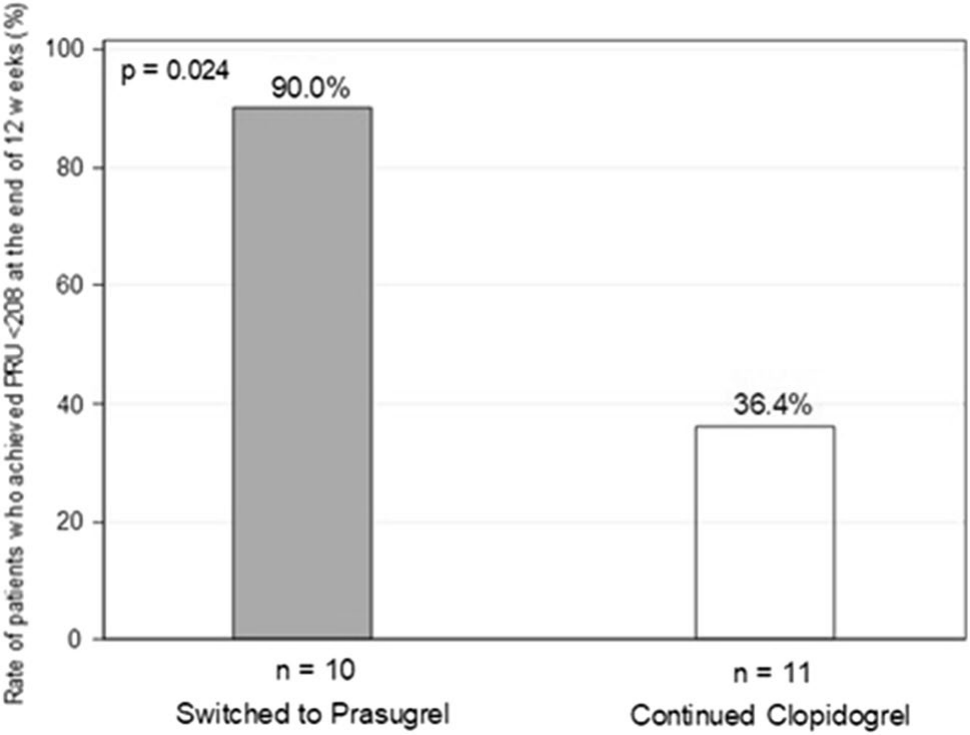
Rate of patients who achieved PRU < 208 at the end of 12 weeks. In “Switched to Prasugrel” group, one patient was excluded from the denominator because PRU at the end of 12 weeks was not measured. PRU was measured by VerifyNow described in “Methods”

As shown in Fig. 3, the rate of patients who achieved PRU < 208 at the end of week 12 after randomization was significantly higher in Switched Group (9/10 patients, 90.0%), relative to Continued Group (4/11 patients, 36.4%) (*P* = 0.024). Among 11 patients assigned to Switched Group, one was excluded from the denominator because PRU at the end of week 12 was not measured.

### Secondary endpoint

**Fig. 4:**
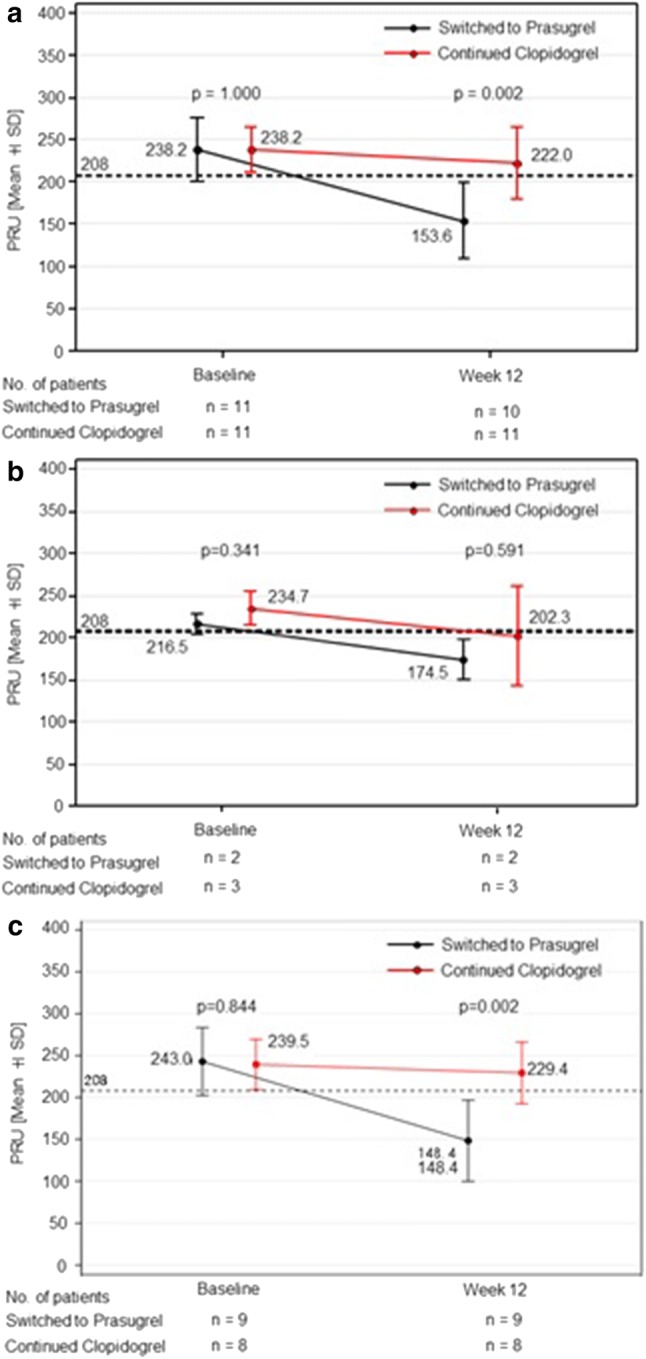
Change from baseline to week 12 in PRU per treatment group. **a** PRU were measured at baseline and week 12. The mean of PRU on all patients of “Switched to Prasugrel” and “Continued Clopidogrel” were compared. **b** The mean of PRU on extensive metabolizer (EM) patients of “Switched to Prasugrel” and “Continued Clopidogrel” were compared. **c** The mean of PRU on intermediate metabolizer (IM) + poor metabolizer (PM) patients of “Switched to Prasugrel” and “Continued Clopidogrel” were compared

PRU at baseline had been 238.2 ± 26.7 in Continued Group and 238.2 ± 37.9 in Switched Group (*P* = 1.000), though at week 12 changed to 220 ± 42.8 and 153.6 ± 44.8, respectively (*P* = 0.002), demonstrating significant reduction in PRU in Switched Group (Fig. 4a). When examined according to CYP2C19 polymorphism, PRU at baseline in EM Group had been 234.7 ± 19.9 in Continued Group and 216.5 ± 12.0 in Switched Group (*P* = 0.341), at week 12 changed to 202.3 ± 60.0 and 174.5 ± 22.3, respectively (*P* = 0.591), with no difference between these groups (Fig. 4b). In non-EM Group, as well, no difference was observed in PRU at baseline between Continued and Switched Groups (239.5 ± 30.0 and 243.0 ± 40.4, *P* = 0.844), however, at week 12 changed to 229.4 ± 36.9 and 148.4 ± 48.4, respectively (*P* = 0.002), demonstrating significant reduction in PRU in Switched Group (Fig. 4c).

No cardiovascular events were observed in either group during the study period. Bleeding events (BARC Type 1) were observed in both treatment groups (2 cases, including nosebleed and subcutaneous bleeding in Continued Group, 1 case of subcutaneous bleeding in Switched Group). Other adverse events were experienced in 5 out of 11 patients in Continued Group (including one serious adverse event: vertebral compression fracture), and in 4 out of 11 in Switched Group (including one serious adverse event: influenza).

## Discussion

This study enrolled patients who had been chronically with DAPT (aspirin + clopidogrel) after PCI with coronary stent implantation. PRU were assessed for all patients, and those with PRU ≥ 208 were randomly assigned to Continued Group or Switched Group to evaluate PRU at the end of week 12 of treatment. The rate of patients who achieved PRU < 208 at week 12 was significantly higher in Switched Group relative to Continued Group (90.0% vs. 36.4%). Based on the results of the CYP2C19 genotype test for all patients in this study, the proportions with EM, IM, and PM were 35.4% (34 patients), 52.1% (50 patients), and 12.5% (12 patients), respectively. Compared among the enrolled patients, non-EM Group showed a higher proportion in PRU ≥ 208. Furthermore, there were no cardiovascular or bleeding events with statistically significant differences in either group during the study period.

### Examined PRU in patients with long-term DAPT

All patients in this study had been chronically with DAPT (aspirin + clopidogrel) for approximately 5 years (median DAPT duration). About 60% had a DAPT score of ≥ 2, indicating that long-term DAPT treatment was generally appropriate. Few studies have evaluated the antiplatelet effects based on PRU in patients with long-term DAPT duration over 5 years. This study investigated these patients using the PRU cutoff value of 208, at which the risk of cardiovascular events showed significantly higher in the ADAPT-DES study [11, 12], and found a high proportion (a quarter patients) were high-risk with PRU ≥ 208. Since this study was conducted in a small number of patients, these results should be confirmed in a study of larger size.

### Switching between P2Y_12_ inhibitors in patients with long-term DAPT

In our previous study, the CONTINUE VERSUS SWITCH-Kyushu study [25], patients after PCI with coronary stent implantation were treated with prasugrel for 2 weeks, and randomly assigned into either continuing prasugrel or switched to clopidogrel, for the assessment of PRU at week 4 from randomization. The rate of patients who achieved PRU < 208 at week 4 in groups continued prasugrel and switched to clopidogrel was 94.7% and 66.1%, respectively. This study found that the rate of patients who achieved PRU < 208 at week 12 in Switched Group was significantly higher relative to Continued Group (90.0% vs. 36.4%). It is also found that even those with long-term DAPT could reduce their PRU by switching from clopidogrel to prasugrel. The reason why the patients in Switched Group did not achieve PRU < 208 is a future consideration.

Due to the difference in timing of PRU measurement between the ADAPT-DES study and this study, the risk of cardiovascular events in patients with PRU ≥ 208 assessed after long-term DAPT has not been established. It is not rare in healthcare settings that long-term DAPT is required for patients who had placed a first-generation drug-eluting stent, patients with complex PCI, and judged to have a high risk of cardiovascular events, based on such indicators as DAPT score. Although PRU measurement is not generally practiced at all medical facilities, switching from clopidogrel to prasugrel under appropriate risk management based on PRU could be an option for treatment in patients with a potentially high risk of cardiovascular events.

### Examined CYP2C19 polymorphism and PRU in patients with long-term DAPT

This study has established that PRU at baseline in IM and PM patients was significantly higher compared to that in EM patients, even after long-term DAPT (aspirin + clopidogrel). In addition, non-EM Group with PRU ≥ 208 in Switched Group steadily decreased PRU compared to Continued Group, with less affected by CYP2C19 polymorphism. Recent meta-analyses studies have reported the frequency of cardiovascular events higher in patients treated with clopidogrel who carry CYP2C19 loss-of-function alleles, compared to patients without the alleles [15, 16]. Additionally, a personalized approach of selecting appropriate antiplatelet agents for patients with CYP2C19 loss-of-function alleles is reported to have reduced the incidence of cardiovascular events [26, 27]. Similar to PRU measurement, it is difficult to perform CYP2C19 genotype testing at all medical facilities. However, prevalence of PM in the Japanese population is approximately 20%, it may be preferable to select an appropriate P2Y_12_ inhibitor after CYP2C19 genotype test, to reduce the cardiovascular events after DAPT.

### Switching between P2Y_12_ inhibitors and bleeding events

Risk of bleeding events is a recent topic, often mentioned with respect to antiplatelet agents in the field of PCI. The PRASFIT-ACS and PRASFIT-Elective studies conducted in Japan reported that risk of bleeding events was female sex, age ≥ 75 years, and weight ≤ 50 kg [28]. The present study also found that patients with PRU ≥ 208 after long-term DAPT (aspirin + clopidogrel) included more females, more with advanced age and lower body weight, compared to those with PRU < 208. After switching treatment from clopidogrel to prasugrel in these patients, no major bleeding events were observed, and the incidence of minor bleeding events was similar to that in Continued Group. While further consideration is required, the Japanese large-scale studies in patients with cardiac or brain diseases demonstrated the equivalent safety with respect to the incidence of major bleeding events between clopidogrel 75 mg daily prasugrel 3.75 mg daily [17, 19, 29].

The overseas guidelines recommend an early switch from DAPT to single antiplatelet therapy (SAPT) for patients with risk of bleeding events, and some clinical research is ongoing in Japan to investigate the appropriateness of shortening DAPT duration. When changing treatment to SAPT, there are more than a few cases to consider the option of SAPT with a thienopyridine drug, since there are multiple reports on aspirin associated with risk of gastrointestinal and intracranial bleeding [30–32]. In such cases, to reduce future incidence of cardiovascular events, it may be necessary to select an antiplatelet agent in which PRU exhibits an appropriate therapeutic window.

## Limitation

In this study, the target number of patients suggested by the rationale could not be collected in this study period. The reasons for this include the small number of patients who had been with long-term DAPT (aspirin + clopidogrel) in each site, different distribution of CYP2C19 polymorphism compared to previous reports, and lower than assumed PRU in patients with CYP2C19 polymorphism. However, the study’s primary endpoint was achieved. In this study, the PRU cutoff value was defined as 208 according to previous studies for patients within 2 years after PCI [11, 12]. Moreover, Asian patients enrolled in the previous studies were very few. Further investigation is required to determine the optimal cutoff value in Japanese patients more than 2 years after PCI.

## Conclusion

For patients with long-term DAPT (aspirin + clopidogrel) after PCI with coronary stent implantation, switching from clopidogrel to prasugrel resulted in stable reduction in PRU, regardless of CYP2C19 polymorphism. Further examination is required to investigate whether or not the results of this study lead to a reduction in cardiovascular events.
